# Photocatalytic degradation of 4-chlorophenol by UV/H_2_O_2_/NiO process in aqueous solution

**DOI:** 10.1186/1735-2746-9-12

**Published:** 2012-11-27

**Authors:** Roya Alimoradzadeh, Ali Assadi, Simin Nasseri, Mohammad Reza Mehrasbi

**Affiliations:** 1 Department of Environmental Health Engineering, Zanjan University of Medical Sciences, Zanjan, Iran; 2 Department of Environmental Health Engineering, School of Public Health and Center for Water Quality Research, Institute for Environmental Research, Tehran University of Medical Sciences, Tehran, Iran

**Keywords:** 4-Chlorophenol, UV light, Nickel oxide, Hydroxyl radicals, Degradation rate

## Abstract

The removal of 4-chlorophenol from aqueous phase continues to be an important environmental issue. In this work, the photochemical oxidation of 4-chlorophenol in aqueous solutions in a batch reactor using ultraviolet irradiation, hydrogen peroxide and nickel oxide was studied. The efficiency of the system was evaluated with respect to reaction time, pH, feed concentration of reactants, catalyst load, light intensity, and the reaction rate constant. The concentrations of 4-chlorophenol and chloride ions were determined by high performance liquid chromatography and ion chromatography, respectively. Pure nanosized nickel oxide was characterized by X-ray diffraction and scanning electron microscopy. The results showed that the optimum conditions (the complete 4-chlorophenol removal (100%) at 60 min) were obtained at a neutral pH, with 0.2 mol/L H_2_O_2_, and 0.05 g/L of nickel oxide. However, no pH effects were observed in the range of 4–10. Analytical profiles on 4-chlorophenol transformation were consistent with the best line fit of the first-order kinetics. Moreover, the degradation rate constant increased with both UV light intensity and decreasing initial concentration of 4-chlorophenol. Finally, the results of mineralization and chloride ions studies indicated that dechlorination was better accomplished but more time was required to completely mineralize 4-chlorophenol into water and carbon dioxide.

## Introduction

Nowadays, a high number of organic contaminants are resistant to conventional chemical and biological treatments. Therefore, the sustainability approach in water sources management includes enhanced demands on new wastewater treatment technologies in order to reduce the negative impacts on the water bodies and to facilitate recycling and reuse of wastewater [1].

Advanced oxidation processes (AOPs) constitute a collection of established treatment technologies which rely on the formation of hydroxyl radical to affect the destruction of emergency pollutants [2-4]. During the last ten years, heterogeneous photocatalytic AOP is placed in the forefront of most researches due to its high efficiency in total degradation of pollutants, non-selectively and generation of benign products [3,5,6]. Photocatalytic oxidation processes deal with photoactivated metal oxides as semiconductors to remove contaminants in aqueous environment [7].

The photocatalytic mechanism begins when a photon with energy *hv* matches or exceeds the band gap energy of the semiconductors. Conduction electrons are promoted from the valance band into the conduction band (CB), leaving a hole behind. The hole can either oxidize a compound directly or react with electron donors like water to form OH radicals, which in turn react with pollutants [8-10].

TiO_2_ is the main semiconductor used due to its ready availability, low cost, activity under a wide range of pH, efficiency and long-term stability [3,11]. The main drawback of TiO_2_ is based on the economy of the extensive use for large-scale facilities, and, in some cases, on the low mineralization level achieved, requiring a final polishing stage [12]. There is renewed effort to study for more reliable semiconductors [1,13].

Transition metal oxides have proved to be active in the photocatalytic reactions of the elimination of chlorophenols and its derivatives [14-16]. In this study, NiO was selected as the alternative semiconductor. Since NiO is an important transition metal oxide widely used as a catalyst with extraordinary electrical, thermal, catalytic, and redox properties [17,18]. Most attracting features of NiO are excellent durability and electrochemical stability. Reported bandgap energy value for the nickel oxide is in the range of 3.4-3.8 eV. This suggests that the optical transition in NiO takes place through direct inter-band transition [19]. Also, NiO can act as a promoter for the generation of OH radicals [20].

Chlorinated phenols are listed as priority pollutants since they are toxic, recalcitrant, and suspected carcinogen and mutagen to living, chlorophenols (CPs) posing serious ecological problems [21]. Environmental issues with these chemicals occur from industrial wastewater, such as petroleum refining, and production of pesticides, paint, plastic, resin, textile, iron, solvent, pharmaceutics and wood preserving chemicals [22-26]. 4-chlorophenol (4-CP) has attracted interests owing to its direct relevance to environment and informative photochemical behaviors [27].

Different AOPs removing phenolic compounds have been reported, such as UV/H_2_O_2_[28], UV/catalyst [29], photo-Fenton process [30], MW/NiO [31], UV/H_2_O_2_/TiO_2_[32,33], O_3_/ZnO [34] and UV/O_3_/TiO_2_[12]. Despite extensive research, robust and economical treatment of the 4-CP has still to be implemented, and, there is a continued need to develop effective systems. The major objective of this study was to examine the efficiency of UV/H_2_O_2_/NiO system. The effects of pH, concentration of H_2_O_2_, and the amount of NiO photocatalyst, on 4-CP degradation rate were also evaluated.

## Materials and methods

4-CP (purity 99%) and hydrogen peroxide (30%, w/v) reagents were purchased from Merck Co. Nickel oxide nano powder (99.8% purity and <50 nm particle size) was obtained from Sigma–Aldrich. Other chemicals were of analytical grade and were used without further purification. Deionized and doubly distilled water was used throughout this study. The pH of solution was adjusted using NaOH and H_2_SO_4_, where needed.

### Experimental set-up

**Figure 1:**
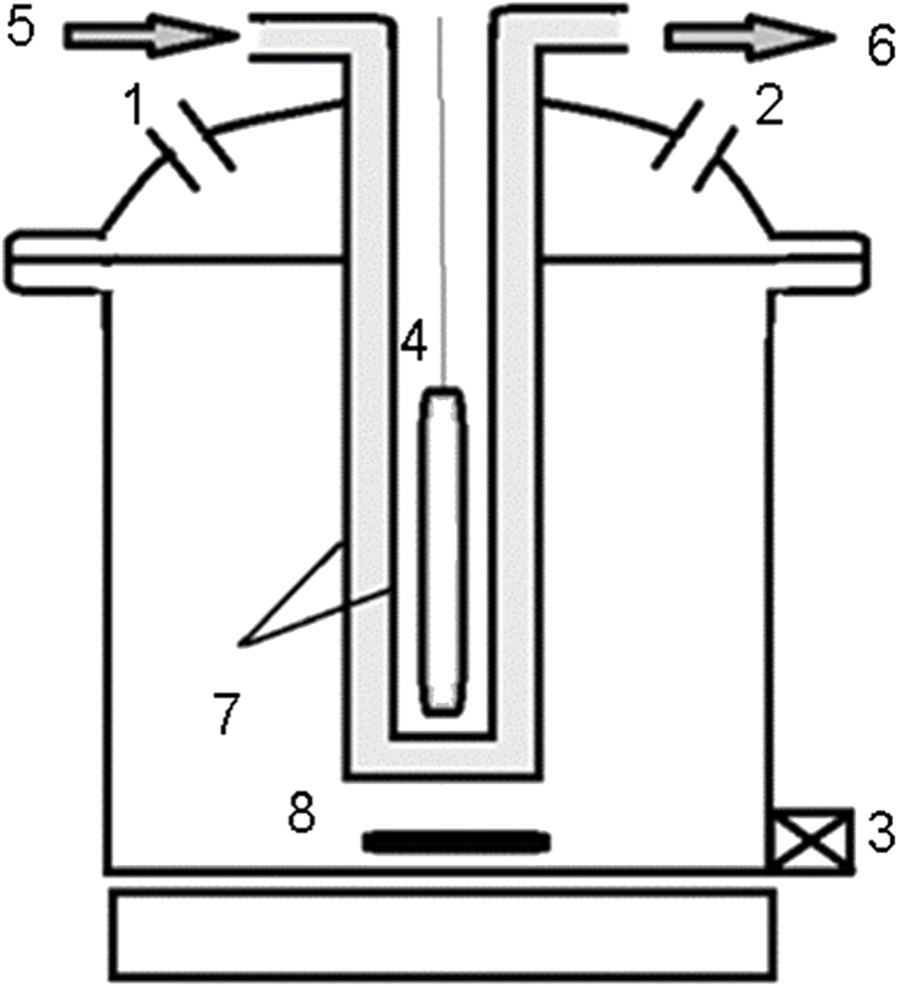
Schematic of the experimental set-up: 1 and 2: chemical feed; 3: sampling port; 4: UV emitting system; 5–6: water inlet and outlet; 7: double-walled cooling system; 8: stirrer.

The photocatalytic experiments were performed in a batch reactor. The schematic experimental details of reactor are shown in Figure 1. It consisted of a cylindrical glass reactor (2 L total volume) with an inner diameter and height of 11 cm and 16 cm, respectively. In each experiment, the reactor was filled with 1000 mL of an aqueous solution of 4-CP with predetermined pH value. Irradiation was achieved by using UV lamp of 150 W (medium-pressure lamp) with main emission wavelength at 247 nm. The UV lamp was located vertically in the center of reactor within a double-wall cooling system. The reaction chamber was filled with the mixture, which was placed between the reactor wall and UV lamp system.

### Procedure

The reactor was filled with the reaction mixture containing constant concentration of 100 mg/L 4-CP to simulate a high loaded industrial wastewater. The reaction was performed at neutral pH except for a few runs to evaluate pH effects on the reaction rate. For runs of UV/H_2_O_2_/NiO system, the pH value of solution was adjusted at the desired value before start-up, and then a given amount of NiO was added. The nanocatalyst was mixed very well with 4-CP before the addition of given volume of hydrogen peroxide. The time at which UV lamp was turned on was considered time zero. The initial concentrations of H_2_O_2_ and NiO varied in the range of 0.005-0.4 mol/L and 0.025-0.2 g/L, respectively.

### Analysis

Samples were taken from the sample port of the reactor at predetermined time intervals. Potential reactions with hydrogen peroxide and hydroxyl radicals in the samples were quenched by using 6 M NaOH solution. The samples were filtered by syringe membrane filter (0.45 μm pore-size) to remove NiO particles. Analyses of initial and remaining concentrations of 4-CP were determined with a Knauer HPLC instrument with a reversed phase C18 column (Erouphere 250 × 4.6 mm). The injected volume of reaction solution was 20 μL. The mobile phase was prepared by methanol and deionized water (containing 2% acetic acid) in 52/48 (V/V) ratio at a flow rate of 1 mL/min. The UV detector was set to 278 nm [35].

Ion chromotoghraphy (881 Compact IC pro, Metrohm) equipped with a Metrosep ASUPP4 column (250 × 4.0 mm) was employed for the analysis of chloride ion concentrations in aqueous solution using NaCl as a standard. The mobile phase of a mixture of 2.5 mM Na_2_CO_3_ and 2.4 mM NaHCO_3_ was used at a flow rate of 0.7 mL/min. The volume of each sample was 20 μL. The total organic carbon (TOC) was measured by means of a Dohrmann DC-190 (Rosemount Analytical Inc., Santa Clara, CA, USA) high-temperature TOC analyzer based on the combustion-infrared method.

Calibration curve at six concentration levels were prepared from working solutions containing 4-CP in the range of 0.1-100 mg/L (R^2^ = 0.999). Detection limit of this method for determination of 4-CP was 0.02 mg/L and the relative standard error (RSD) did not exceed 0.2% based on triplicate.

X-ray diffraction (XRD) measurements were performed using a diffractometer (GNRMPD 3000, Italy) with Cu K_α1_ radiation (*λ* = 1.5405 Å) at 40 kV and 30 mA with a scanning speed in 2*θ* of 4-70^º^ with 1 s counting time. The crystallite size of nickel oxides was estimated using the Scherrer equation. The surface of NiO particles was observed with a scanning electron microscopy (SEM) (VEGAII, TESCAN electron microscopy).

## Results

### Characterization of NiO

**Figure 2:**
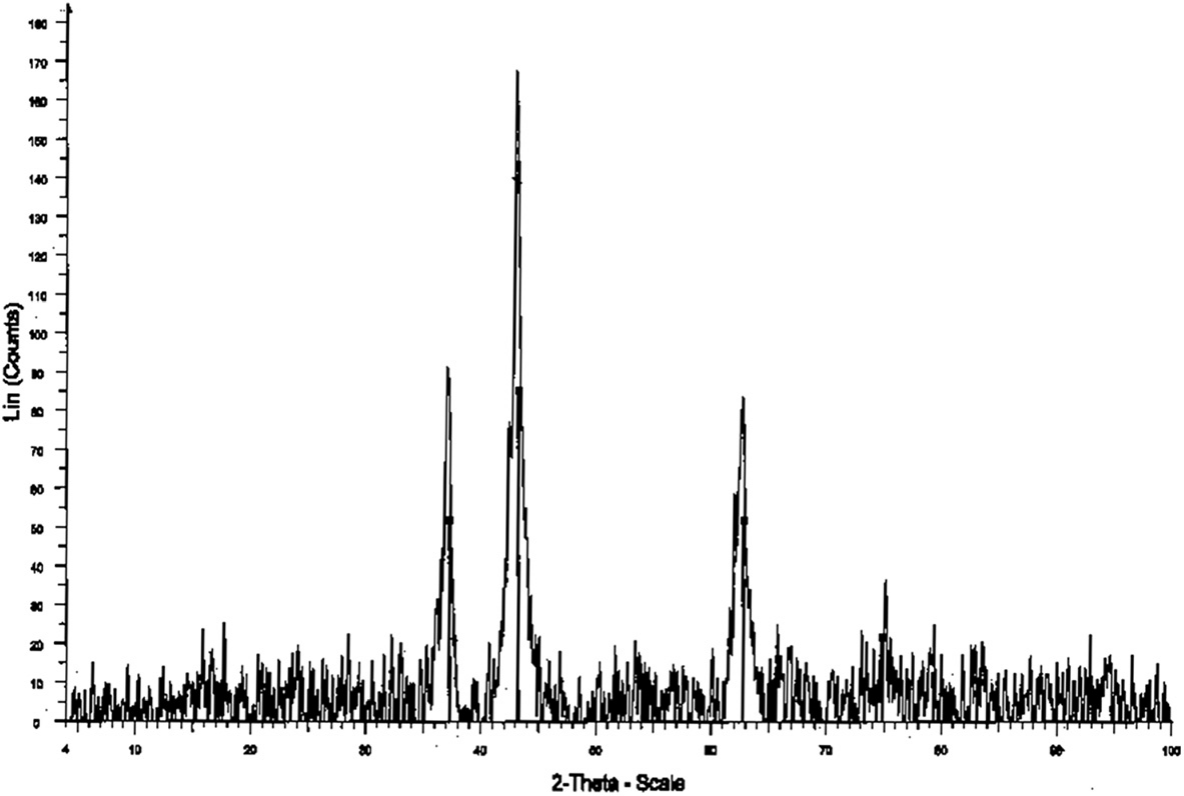
XRD pattern of NiO nanoparticles.

**Figure 3:**
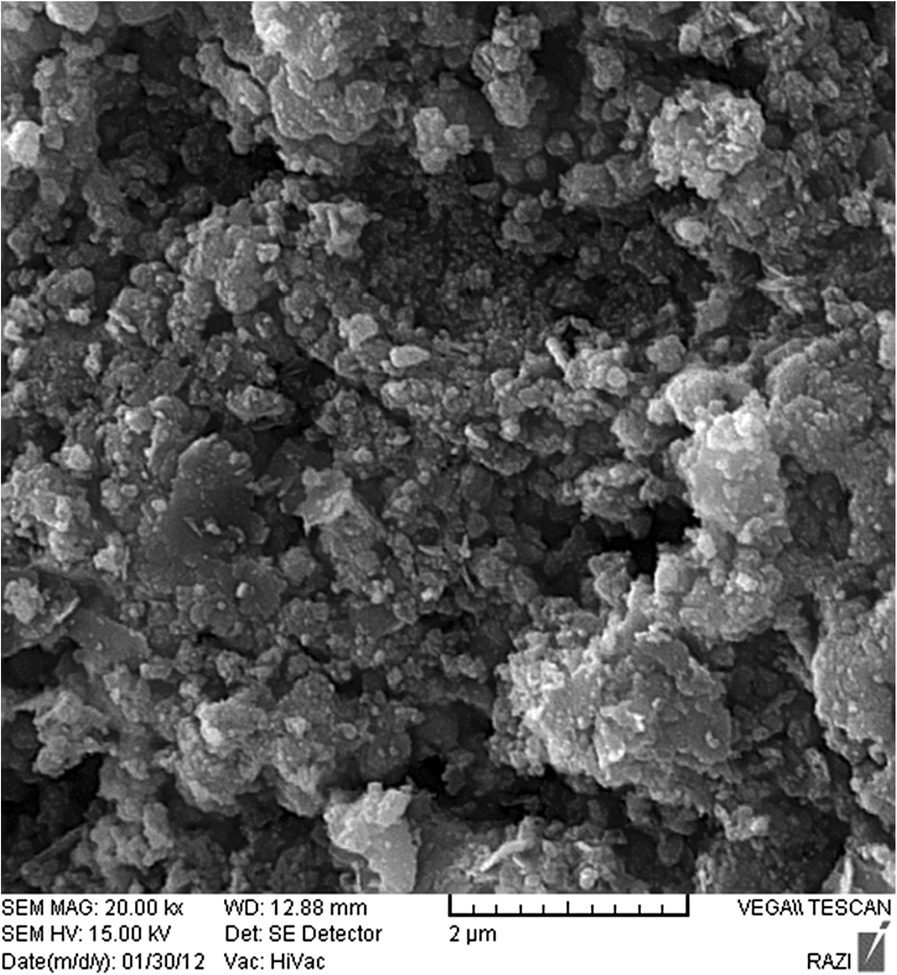
The SEM microphotograph of NiO particles.

The XRD pattern of NiO showed the presence of three peaks (Figure 2). The second peak (2θ = 43.1), is an indicator of nickel oxide form. Moreover, the average particle size of NiO are determined to be approximately about 100 nm from each peak in the XRD pattern. Figure 3 shows the SEM images of nanoparticles. It demonstrates almost uniform morphologies with size in the range of 53–117 nm.

### Blank experiments

**Figure 4:**
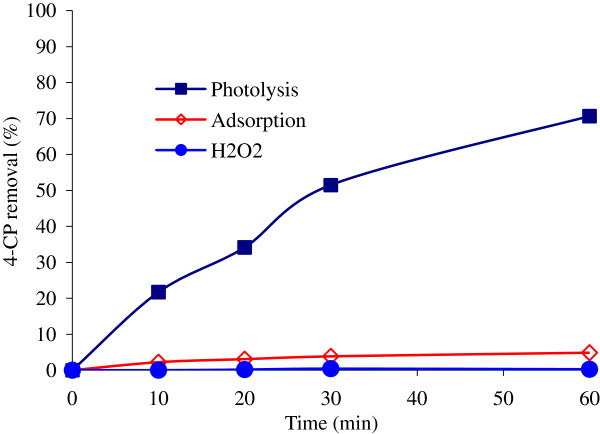
**Preliminary experiments of 4-CP removal (Co = 100 mg/L; H**_**2**_**O**_**2**_ **= 0.2 mol/L; NiO = 0.05 g/L;pH = 7).**

Before conducting photocatalytic oxidation runs, several blank experiments were performed in order to validate the removal route of 4-CP through the mechanisms other than photocatalytic reactions. The adsorption of 4-CP from the aqueous solution was found to be less than 4.5% in the dark system during 60 min. However, all parts used in the reactor was glassware to minimize the effect of adsorption. The role of UV alone as function of time showed moderately change in the concentration of 4-CP. Also, it was found that H_2_O_2_ could not remove 4-CP alone. Figure 4 shows the profile of 4-CP removal separately subjected to adsorption, photolysis and H_2_O_2_.

### Initial H_2_O_2_ concentration effects

**Figure 5:**
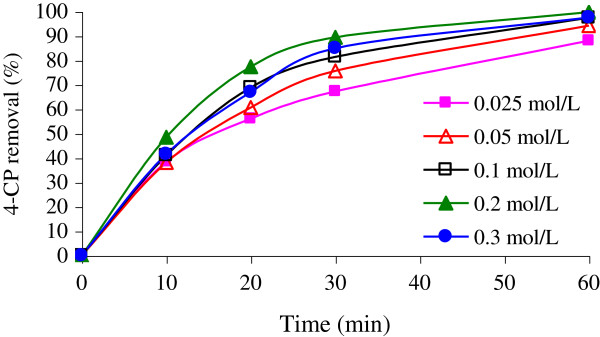
**The effect of initial concentration of H**_**2**_**O**_**2 **_**in degradation of 4-CP(Co = 100 mg/L; pH = 7; NiO = 0.05 g/L).**

Oxidant concentration greatly affects the rate of degradation. Figure 5 compares the photodegradation of 100 mg/L 4-CP under various concentrations of H_2_O_2_ (0.025-0.3 mol/L) at neutral pH. By addition of H_2_O_2_, the complete 4-CP removal (100%) was obtained up to 0.2 mol/L at 60 min, whereas at higher H_2_O_2_ concentration at the same irradiation time, the 4-CP removal decreased. At these operating conditions, the optimum H_2_O_2_ concentration was selected at a 0.2 mol/L.

### Effect of NiO dosage on 4-CP removal

**Figure 6:**
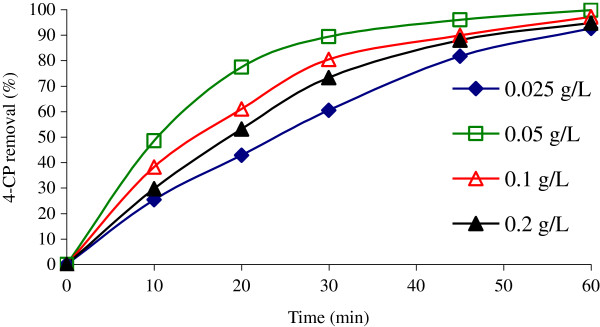
**4-CP degradation as a function of the NiO dosage (Co = 100 mg/L; H**_**2**_**O**_**2**_ **= 0.2 mol/L; pH = 7).**

To determine the optimal NiO amount, various dosages of catalyst (0.025-0.2 g/L) were used to mix with constant concentration of 4-CP. Figure 6 shows that the addition of NiO increased the efficiency of UV/H_2_O_2_/NiO system for 4-CP oxidation. When NiO dosage increased to 0.05 g/L, removal of 4-CP increased to 100%. However, further addition of the NiO dosage resulted in a slight decrease in 4-CP removal to less than 95%, even when the concentration of the catalyst was doubled. The optimal amount of NiO was confirmed to be 0.05 g/L for 4-CP degradation.

### Influence of solution pH

**Figure 7:**
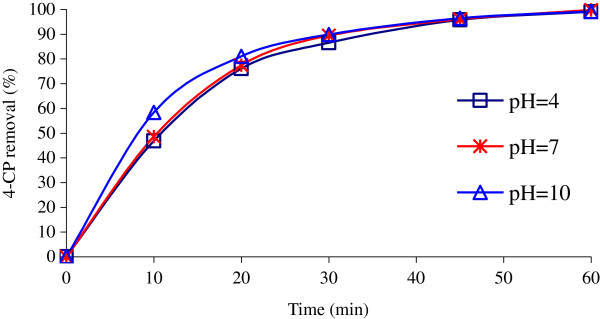
**4-CP degradation as a function of the pH value (Co = 100 mg/L; H**_**2**_**O**_**2**_ **= 0.2 mol/L; NiO = 0.05 g/L).**

It is clearly shown in Figure 7 that the performance of UV/H_2_O_2_/NiO system is independent of pH and no significant effect was observed in the range of 4–10. Although, little decreasing was observed in acidic pH. The best results were obtained at neutral pH, where that 4-CP completely disappeared in less than 45 min irradiation.

### Effect of the initial 4-CP concentration

**Figure 8:**
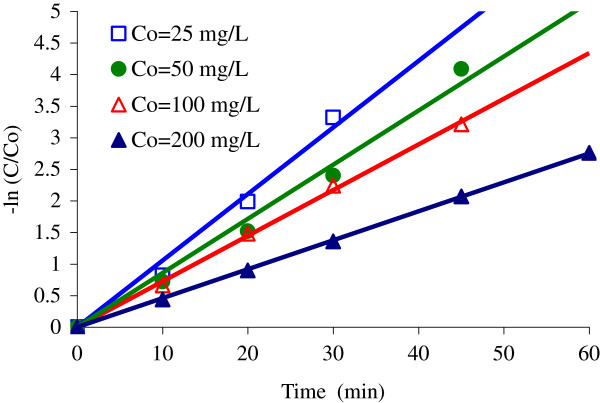
**Effect of initial 4-CP concentration on constant reaction rate (k) using UV/H**_**2**_**O**_**2**_**/NiO process (H**_**2**_**O**_**2**_ **= 0.2 mol/L; pH = 7; NiO = 0.05 g/L)**.

The reaction constant rate of 4-CP at different initial concentrations (Co = 25-200 mg/L) under constant molar ratio of H_2_O_2_ to 4-CP (257/1) are compared in Figure 8. The photodegradation reaction kinetic of 4-CP can be described by a modified Langmuir–Hinshelwood model according to the reports [32,36]. It basically relates the degradation rate (*r*) and the concentration of 4-CP (*C*), which is expressed as follows:

(1) $$r=-\frac{dC}{dt}=\frac{k_{r}K_{\text{ad}}C}{1+K_{\text{ad}}C}$$

Where *k*_r_ is the intrinsic rate constant and *K*_ad_ is the adsorption equilibrium constant. When the adsorption is relatively low and/or the concentration of organic compound is low, Eq. (1) can be simplified to the first-order kinetics with an apparent rate constant (*k*):

(2) $$\text{ln}\left(\frac{C}{Co}\right)=-kt$$

A plot of − ln(*C*/*C*o) versus reaction time *t* yields a straight line, and the slope is the apparent rate constant (*k*). The removal of 4-CP decreased with increasing Co under the conditions studied; however, the total amount of 4-CP degraded actually increased. It was also confirmed in this study that 4-CP photodegradation using UV/H_2_O_2_/NiO system follows the first-order kinetics. The apparent rate constants (*k*) were 0.105, 0.085, 0.072, and 0.045/ min at Co = 25, 50, 100, and 200 mg/L, respectively.

### Effect of UV light intensity

**Figure 9:**
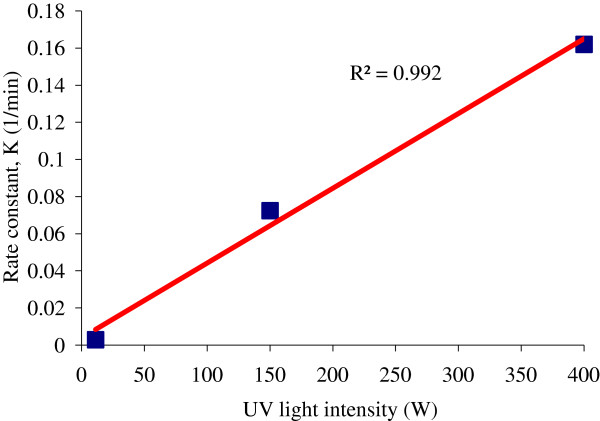
**Relationship between UV light intensity and first-order constant (k) using UV/H**_**2**_**O**_**2**_**/NiO process (Co = 100 mg/L; H**_**2**_**O**_**2**_ **= 0.2 mol/L; NiO = 0.05 g/L; pH = 7)**.

The effect of UV light intensity (11–400 W) on 4-CP destruction was evaluated in the presence of 0.05 g/L NiO. Figure 9 indicates that all the reactions followed the first order kinetics. In the UV/H_2_O_2_/NiO process, the rate constants with a light intensity of 11, 150, and 400 W were 0.003, 0.072, and 0.162/ min, respectively.

### 3.8. Mineralization of 4-CP

**Figure 10:**
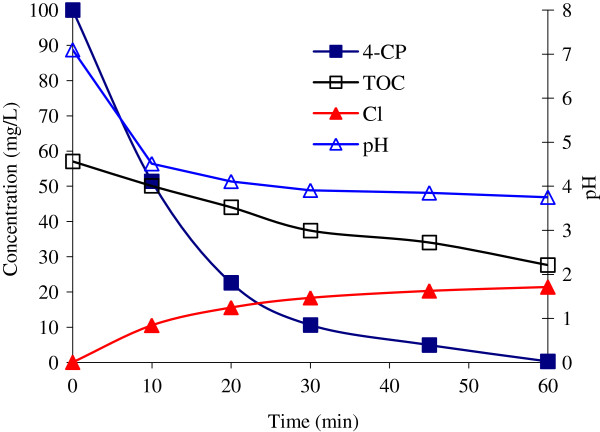
**4-CP degradation and variation of TOC, Cl**^**-**^**ions, and pH profiles in the UV/H**_**2**_**O**_**2**_**/ NiO process (Co = 100 mg/L; H**_**2**_**O**_**2**_ **= 0.2 mol/L; NiO = 0.05 g/L; pH = 7.1)**.

Figure 10 illustrates the mineralization profile of 4-CP by variations of TOC, pH and increase of Cl^-^ concentrations. About 100% 4-CP was removed after 60 min reaction. At that time, the stoichiometric coefficient (mol H_2_O_2_/ mol 4-CP) was 257. However, approximately 48% of TOC was reduced within the same period. Also, a gradual release of chloride ions into the reacting solution was detected during the operation of system. After 60 min of irradiation, the concentrations of Cl^-^ ions reached 21.4 mg/L, whereas the concentration of Cl^-^ atoms initially present in the molecule of 100 mg/L 4-CP was 27.6 mg/L. The depletion of 4-CP and the changes of pH are shown in Figure 10. The value of pH decreased steadily down to 3.91 after 30 min; with further irradiation a pH = 3.75 was reached after 60 min.

## Discussion

In the complete oxidation of 4-CP by UV/H_2_O_2_/NiO process, the color variation could be observed initially; the solution was clear without color, then changed to light brown, and finally to no color again. These observations have been proved with measurement of 4-CP intermediates in the findings of other studies [37].

4-CP removal rate was dependent on the initial concentration of H_2_O_2_ as shown in Figure 5. However, initial H_2_O_2_ concentration had two opposing effects on the removal rate. By addition of H_2_O_2_, the oxidation process enhanced up to a certain point at which H_2_O_2_ started to inhibit the 4-CP oxidation. The most likely explanation for this observation is that H_2_O_2_ acted as a free radical scavenger itself, thereby decreasing the hydroxyl radical concentration [2].

The amount of catalyst is a significant parameter in photocatalytic process [32]. According to the results of 4-CP removal (Figure 6), the NiO dosage of 0.05 g/L reveals comparable performance at 60 min. Several works have proved that the removal rates are strongly affected by the number of active sites and the photo-absorption of the catalyst used. Adequate dosage of the catalyst increases the generation rate of electron/hole pairs; thus, the formation of OH radicals for promoting photodegradation. However, an overdose of the catalyst decreases the UV penetration due to opacity caused by excess catalyst clusters and at the same time increases the scattering effect [3,32].

The UV/H_2_O_2_/NiO process demonstrates that the removal of 4-CP is not affected significantly by pH. As shown in Figure 7, the fastest rate of 4-CP degraded occurs within 10 min under different pH values (degree of conversion obtained 58.4% for pH = 10 but 48.5% for pH = 7). Although little decreasing was observed in acidic pH value. The most likely reason for this observation is the very low substantial loss of NiO to Ni^+2^ at low pH value [16]. The removal rate increases at higher pH value because NiO stability is less disturbed. This provides further support to earlier work that 4-CP can be totally removed in the pH range of 4–10 in 30 min by MW/NiO system [31]. In contrast, the findings of other studies with different catalysts have shown that pH affects significantly the photocatalytic degradation of 4-CP or generation of hydroxyl radical [3,11,29,31].

The reaction rate constant (k) of the photocatalytic process, decreased with increasing the initial concentration of 4-CP (Figure 8). The main reason is that the formation of hydroxyl radical is constant for a given amount of the catalyst. Hence, the available OH radical are insufficient for 4-CP degradation at higher concentrations [38]. Moreover, the higher the 4-CP concentration, the higher concentrations of intermediate products are produced which compete for reacting with OH radicals generated from surface-trapped photogenerated holes [3]. Similar results were observed when TiO_2_ nanotube were used to degrade 4-CP, that the photocatalytic process was rather promising at low 4-CP concentrations which was attributed to adequate of the electron/hole pairs and reactive species formed [38].

The results of this study revealed that UV light intensity has a positive effect on 4-CP removal rate. The light intensity of 400 W had a significant accelerating effect on the rate of 4-CP degradation with rate constant (k) of 54 times higher than the 11 W and 2.25 times higher than 150 W. As shown in Figure 9, an acceptably good linear correlation exists between the first-order constant and light intensity (R^2^ = 0.992). This could be realized by the fact that excitation of every catalyst by light irradiation at any instant can not be possible. But the probability of excitation can be increased by increasing the intensity of light and leads to formation of OH radical. Hence, the improved degradation rate was observed with increasing in the light intensity [22,32,38].

Complete mineralization is the goal in wastewater treatment containing xenobiotics. It is thus important to follow not only reduction of the initial pollutant but also its mineralization into CO_2_, H_2_O, and inorganic ions [9]. The decrease of TOC and the release of Cl^-^ in the removal of 4-CP by NiO showed that the rate of TOC reduction was remarkably slower than that of 4-CP (Figure 10). About 52% of TOC still remained after 60 min irradiation while 4-CP was more dechlorinated and the concentration of Cl^-^ reached reach 80%. This indicates that the chlorine atom was first removed. Because the breaking of C-Cl bond is energetically easier than that of C-OH bond on 4-CP structure [39]. Also, pH values of 4-CP solution decreased dramatically once the solution was irradiated. Then the pH values decreased smoothly with increasing absorbed dose. The reason for the change of pH value could be attributed to two factors. H^+^ is formed when water is irradiated and the Cl^-^ ions were produced during mineralization process. It is obvious that the decrease of the pH value could be attributed to the generation of HCl. The amount of organic Cl^-^ decreases with increasing absorbed dose and thereby the HCl formation rate also decreases [40].

## Conclusion

The obtained results indicate that the UV/H_2_O_2_/NiO process is robust to remove 4-CP with a constant concentration of 100 mg/L by 0.05 g/L NiO and 0.2 M H_2_O_2_ within 60 min. It is also proved in this research that 4-CP removal using this process follows the first-order kinetics. Moreover, results of mineralization and chloride ions studies showed that dechlorination was better accomplished but more time was required to completely mineralize 4-CP into H_2_O and CO_2_. It could be concluded that this process is a novel and effective for the degradation of 4-CP from aqueous phase and appears to be a promising technique for 4-CP remediation.

## Competing interests

The authors declare that they have no competing interests.

## Authors’ contributions

RA, analyzed the Samples and wrote the original research plan of the project and also wrote the first draft of the manuscript. AA, conducted the sampling framework, supervised the methods of this research and wrote the manuscript. SN, co-supervised the project and was involved in the discussion of the results and edited the manuscript. MRM, participated in planning of sample analysis framework and was involved in the discussion of the results and read the manuscript. All authors read and approved the final manuscript.
